# THE FOURTH AGE IN PROSPECT: A STUDY FROM FIVE NATIONS

**DOI:** 10.1093/geroni/igad104.3643

**Published:** 2023-12-21

**Authors:** David Ekerdt, Jaroslava Hasmanova Marhankova, Helene Fung, Shyhnan Liou, Stephan Lessenich

**Affiliations:** University of Kansas, Lawrence, Kansas, United States; Charles University, Prague, Hlavni mesto Praha, Czech Republic; The Chinese University of Hong Kong, Hong Kong, Hong Kong; National Cheng Kung University, Tainan, Tainan, Taiwan (Republic of China); Goethe University, Frankfurt, Hessen, Germany

## Abstract

Higgs and Gilleard (2015) have uniquely theorized the fourth age as a “social imaginary” of deep old age that blends notions of frailty, abjection, and the moral relations of care. This report evaluates the power, reach, and coherence of the fourth age imaginary among older adults in relative good health. In a qualitative design and within samples at five sites (in Czechia, Germany, Hong Kong, Taiwan, and the United States), 138 adults aged 70+ and still living independently discussed what it would mean to be “not independent” in later life. Replies referenced other people in general, specific people, or one’s own potential experience. Pooled across sites, the views of our participants confirm the theorized features of the social imaginary. Participants spoke readily of gateway infirmities heralding frailty and of frailty’s abjection; expressed dread and abhorrence of dependence, some saying that death would be preferable; and were anxious about nursing homes and about burdening others with an obligation to care for them. In all, the bleak but formidable reputation of the fourth age impinges on those living in the third, raising apprehensions about the future. At the same time, perhaps this prospect of unwanted old age can function as adaptive, motivating efforts to maintain bodily and cognitive integrity, to plan for the future, to cultivate support networks, or to savor the present. Fourth-age studies that document the lived experience of frailty and dependence have the potential to undermine the social imaginary and furnish new narratives for facing the future.

